# Supplementary figures and images for: Determining jumping performance from a single body-worn accelerometer using machine learning
Source: PLoS One. 2022 Feb 10;17(2):e0263846. doi: 10.1371/journal.pone.0263846 (PMC8830617; doi:10.1371/journal.pone.0263846)

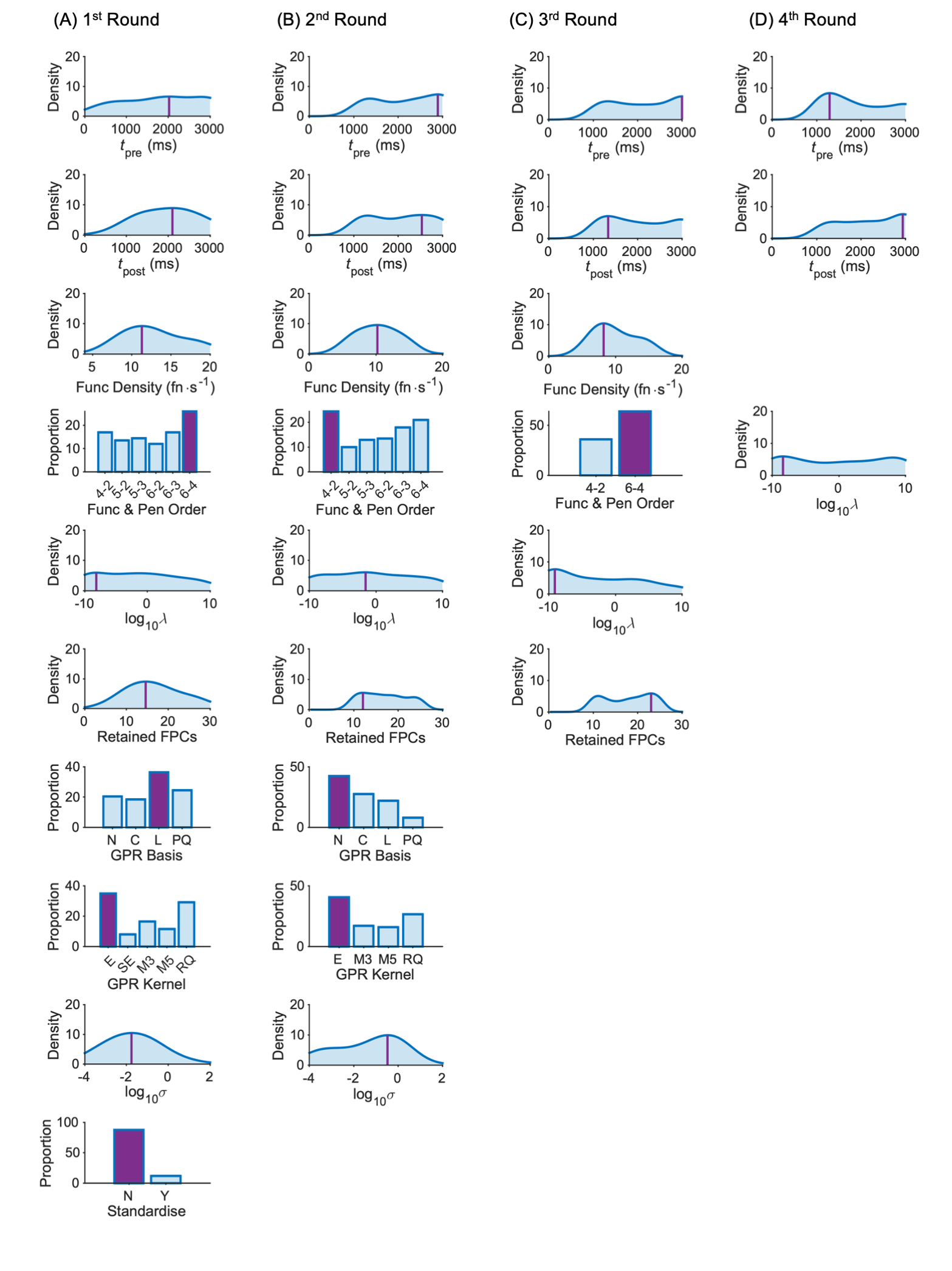

Supplement: S1 Fig — Parameters may be eliminated in successive rounds if there is a clear preference for an optimal value. Alternatively, the range of possible values may be reduced. In doing so, subsequent distributions tend to have more prominent peaks, but not always, as with the time window parameters. Abbreviations. Vertical axes: Proportion = Proportion (%); Density = Probability Density Function × 103. Standardise Axis: N = No; Y = Yes. Regularisation Axis: L = Lasso; R = Ridge. LR Solver Axis: S = SVM; L = Least Squares. (TIF) [file pone.0263846.s001.tif]

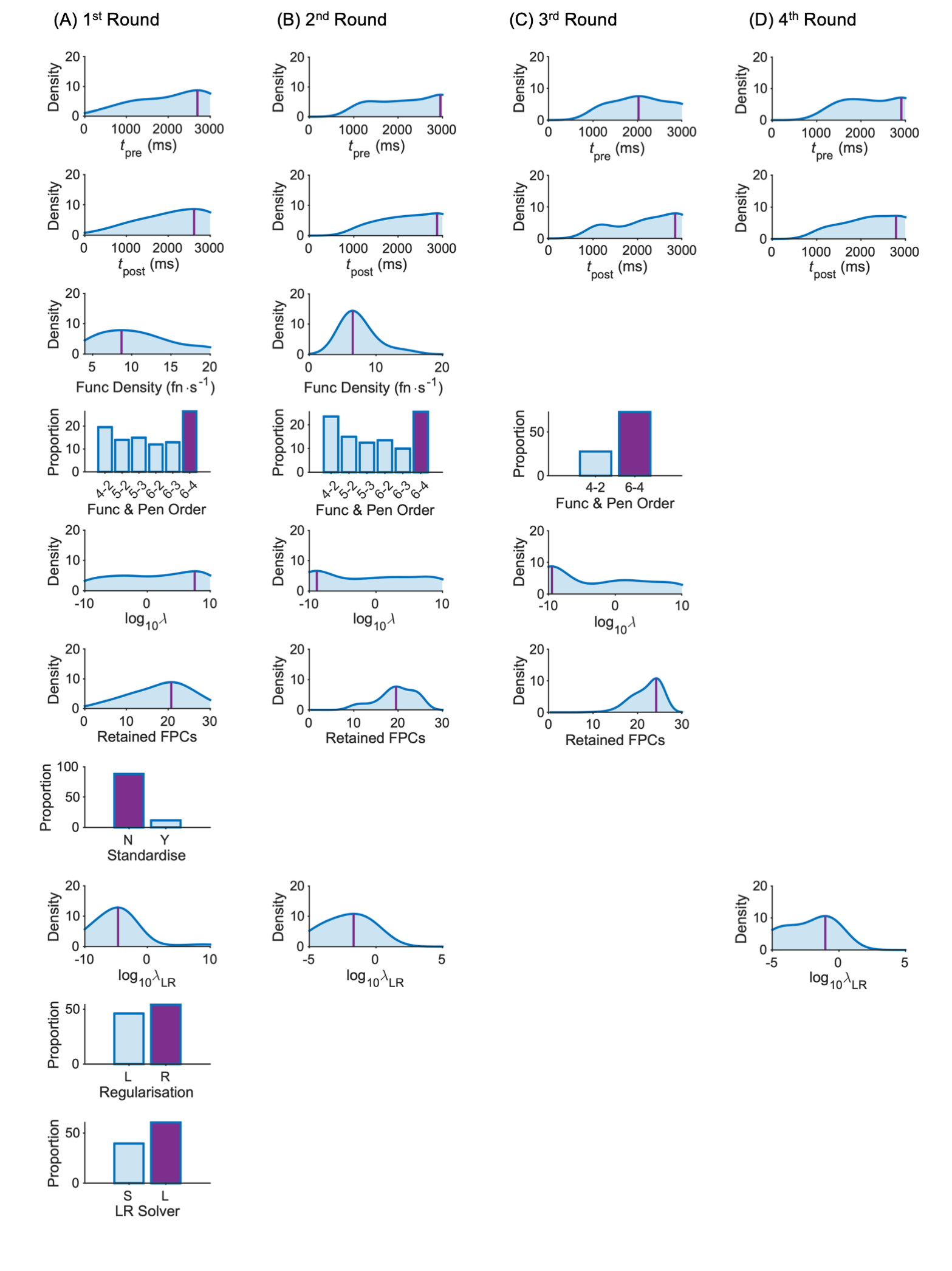

Supplement: S2 Fig — Parameters may be eliminated in successive rounds if there is a clear preference for an optimal value. Alternatively, the range of possible values may be reduced. Abbreviations. Vertical axes: Proportion = Proportion (%); Density = Probability Density Function × 103. Standardise Axis: N = No; Y = Yes. SVM Kernel Axis: L = Linear; G = Gaussian; P = Polynomial. (TIF) [file pone.0263846.s002.tif]

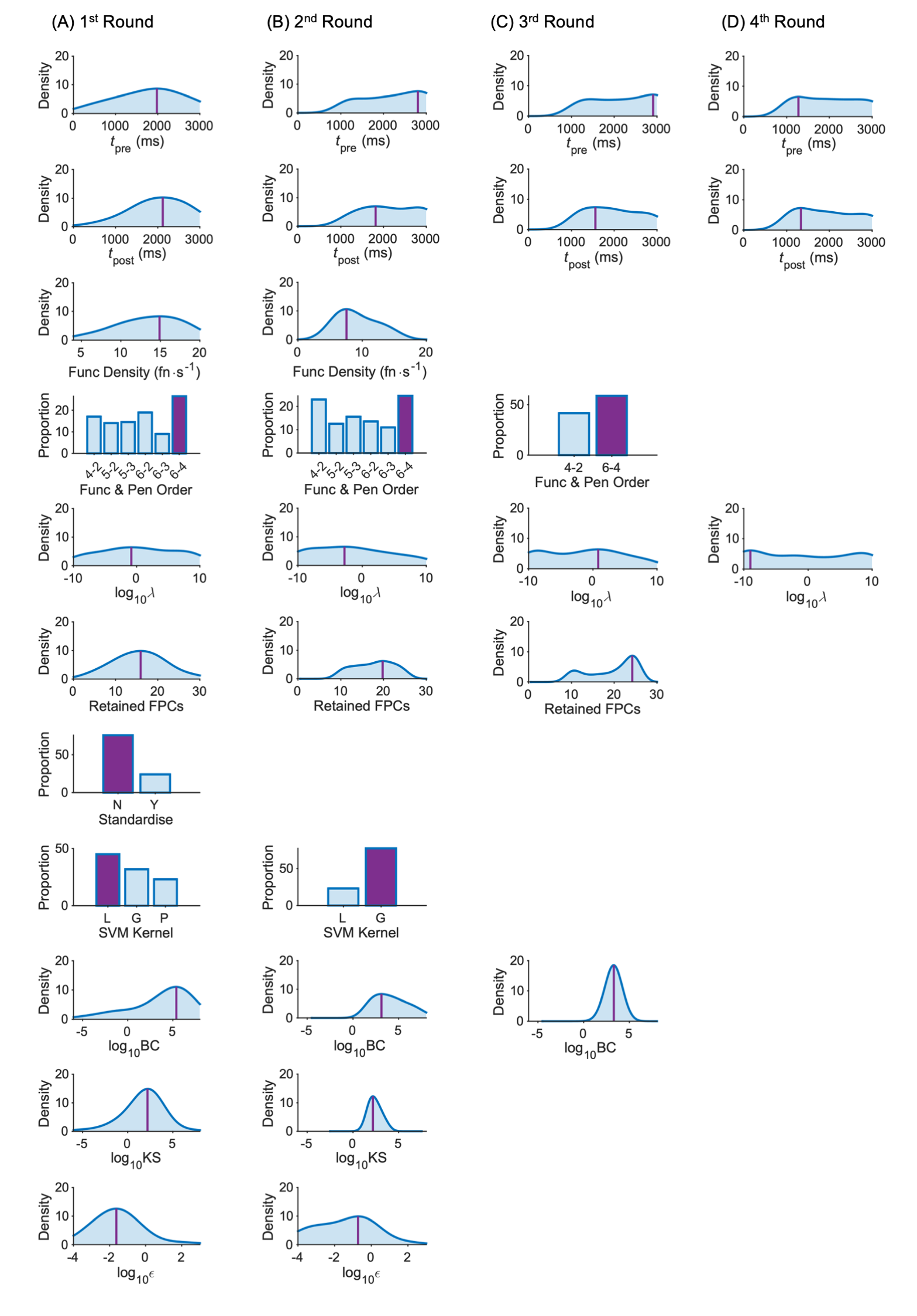

Supplement: S3 Fig — Parameters may be eliminated in successive rounds if there is a clear preference for an optimal value. Alternatively, the range of possible values may be reduced. Abbreviations. Vertical axes: Proportion = Proportion (%); Density = Probability Density Function × 103. GPR Basis Axis: N = None; C = Constant; L = Linear; RQ = Rational Quadratic. GPR Kernel Axis: E = Exponential; SE = Squared Exponential; M3 = Matérn 3/2; M5 = Matérn 5/2; RQ = Rational Quadratic. Standardise Axis: N = No; Y = Yes. (TIF) [file pone.0263846.s003.tif]

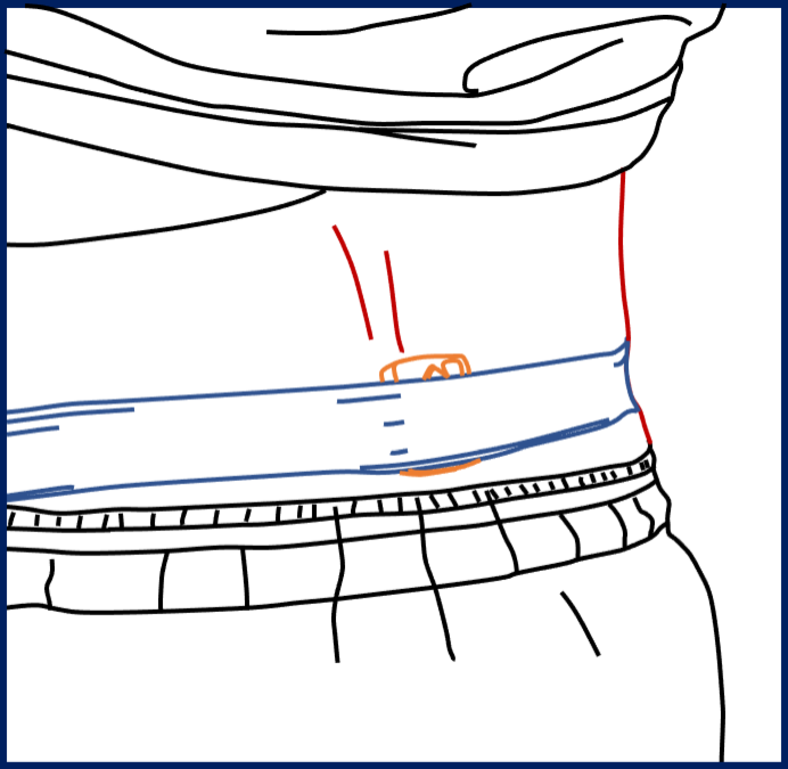

Supplement: S4 Fig — (TIF) [file pone.0263846.s004.tif]
